# Mindfulness-Based Interventions for People with Schizophrenia: A Systematic Review and Meta-Analysis

**DOI:** 10.3390/ijerph17134690

**Published:** 2020-06-30

**Authors:** Ricardo M. Hodann-Caudevilla, Cintia Díaz-Silveira, Francisco A. Burgos-Julián, Miguel A. Santed

**Affiliations:** 1 San Juan de Dios Hospital, 28350 Ciempozuelos, Spain; 2 Psychology Department, Rey Juan Carlos University, 28922 Madrid, Spain; cintia.diazsilveira@urjc.es (C.D.-S.); psico.fburgos@gmail.com (F.A.B.-J.); 3 Personality, Psychological Assessment and Treatment Department, Faculty of Psychology, Universidad Nacional de Educación a Distancia (UNED), 28040 Madrid, Spain

**Keywords:** mindfulness, effectiveness, schizophrenia, meta-analysis

## Abstract

(1) Background: There is increasing interest in the practice of mindfulness-based interventions (MBIs) to treat people with schizophrenia, as evidenced by the publication of different randomized controlled trials (RCTs). However, no meta-analysis of RCTs has been carried out to date with the exclusive inclusion of this type of interventions. (2) Objective: To analyze empirical evidence regarding the effectiveness of MBIs for the improvement of clinical parameters associated with schizophrenia. Method: A systematic review and meta-analysis was conducted of RCTs published in the databases PsycINFO, PubMed, WOS, and Cochrane Library. (3) Results: A total of 10 articles (n = 1094) fulfilled the criteria for inclusion in the review. The analysis of these studies suggests that MBIs combined with standard interventions are able to generate significant improvements in a variety of clinical schizophrenia-related parameters, such as the intensity of overall symptomatology (g = 0.72), positive symptoms (g = 0.32), negative symptoms (g = 0.40), functioning level (g = 1.28), and awareness of illness (g = 0.65). (4) Conclusions: There is evidence that supports the effectiveness and safety of MBIs for the treatment of people with schizophrenia. The results obtained by MBIs are comparable to those obtained by cognitive-behavioral therapy for psychosis. However, given the heterogeneity of the applied interventions and the methodological limitations found in the reviewed trials, the results should be interpreted with caution.

## 1. Introduction

The term mindfulness refers to, at least, two different concepts: (a) a meta-cognitive exercise which involves bringing sustained and intentional attention to experiencing the present moment, while diminishing the emotional and cognitive reactivity generated by the experience or (b) a state of consciousness characterized by the detached observation of one’s thoughts and feelings [1]. Mindfulness mainly comes from the vipassana meditation of Buddhist tradition and plays a central role within the framework of a conceptual and applied system whose ultimate aim is the cessation of suffering [2]. However, it was introduced in the Western world with a secular approach, disassociated from any religious or cultural tradition, as a technique oriented toward promoting the quality of life of people with high stress levels associated with chronic illnesses of a physical type [3]. Over the years, mindfulness has been extended for therapeutic purposes to other health conditions, mainly due to its beneficial effect on people who present psychopathological profiles and somatic diseases [4]. This expansion has taken place through the implementation of several intervention protocols included under the umbrella term mindfulness-based interventions (MBIs).

Amongst the MBIs one can find two seminal protocols, Mindfulness-Based Stress Reduction (MBSR) [3] and Mindfulness-Based Cognitive Therapy (MBCT) [5]. Both are widely used and have remarkable empirical support [6,7]. There is also a broad set of protocols that have been developed from MBSR and MBCT protocols for specific therapeutic purposes. Such protocols usually introduce variations regarding MBSR and MBCT in aspects such as program structure or pedagogical content. However, all MBIs have, as a common point, the systematic training in meditation practices for the development of mindfulness, which is at the heart of the intervention [8]. Other distinctive aspects of MBIs have to do with the way they are administered: usually in group format and through one or more teachers who are not necessarily therapists or mental health professionals [9]. These aspects differentiate MBIs from other therapies—the so-called acceptance-based approaches—in which mindfulness is used as a tool to promote psychological acceptance, but no systematic use of meditation practices is made [10]. This is the case with the Acceptance and Commitment Therapy (ACT) [11] or the Dialectic Behavioral Therapy (DBT) [12], in which mindfulness is taught primarily using short exercises and informal practices which consist of daily activities with a focus on the present moment.

The popularity of MBIs has increased exponentially over the last 30 years, alongside the volume of research conducted on their effectiveness in the treatment of different psychological conditions [13]. Nevertheless, evidence regarding their applicability in schizophrenia is still scarce. This is possibly partly due to a cautious reaction to a few early research papers, which found a relationship between meditation practice and the emergence of psychotic-type symptoms [14,15]. These publications, mostly based on case studies, reported that extended periods of meditation practiced over several consecutive days, in combination with other factors—a history of previous psychotic episodes, sleep deprivation, or the discontinuation of psychiatric medication—could lead to the onset of psychotic disorders. In this regard, authors such as Kuijpers et al. [16] have suggested that, under these conditions, meditation may act as a trigger of symptoms in individuals who have shown vulnerability. Despite this, some studies published in the early 2000s found that the application of therapies which included mindfulness practice resulted in improvements in patients with schizophrenia [17]. Soon after, Chadwick [18] developed the Person-based Cognitive Therapy for distressing psychosis (PBCT), the first intervention specifically designed for people with schizophrenia to incorporate mindfulness as a central element, combined with other therapeutic components derived from cognitive-behavioral therapy (CBT).

CBT is one of the most widespread and empirically supported treatments for schizophrenia [19] used to specifically address psychotic symptoms, such as hallucinations and delusions, and which aims to modify patient held beliefs [20] (pp. 115–118). Although there is a certain amount of controversy regarding its effectiveness in the treatment of schizophrenia [21], on the whole, research yields positive results, especially in the decrease of positive symptoms [22,23,24]. A crucial difference between CBT and MBIs is that the latter do not focus the intervention on the psychotic symptoms per se but rather on the relationship patients establish with psychotic-like experiences. People with schizophrenia learn through mindfulness to abandon their usual response to aversive psychotic experiences—e.g., control efforts and escape-avoidance coping—to adopt a response style fundamentally based on acceptance, which, although counterintuitive, may result in a reduction of the generated distress [25,26].

To our knowledge, four meta-analyses have been published to date on the effectiveness of interventions, which include elements of mindfulness for people with psychosis: Khoury et al. [27], with 13 studies; Cramer et al. [28], with 8 studies; Louise et al. [29], with 10 studies; and Jansen et al. [30], with 16 studies. It is noteworthy that their results are, in general, heterogeneous. Thus, only one reported large effects on overall symptomology [30], and two reported small effects [28,29]. Regarding the characteristic symptoms of schizophrenia, only one found moderate effects on positive symptoms [28] and two found modest effects on negative symptoms [27,30]. However, it is relevant to note that the four meta-analyses cited included in their analyses both MBI’s and acceptance-based approaches (ACT or DBT), which, as mentioned, have significant differences between them. They all included studies in which participants had psychotic symptoms but not necessarily a diagnosis of schizophrenia spectrum disorder (e.g., major depression with psychotic symptoms). These methodological aspects also provide a plausible explanation for the observed variability in their respective results.

The inclusion in the previous meta-analyses of MBI’s and acceptance-based approaches is understandable given that both types of intervention foster the development of mindfulness—by different means—and because of the small number of available studies in which MBI’s were applied to people with schizophrenia. However, we consider that this situation has now evolved, thanks to the recent publication of high-quality studies in this respect. Consequently, we conducted a systematic review and meta-analysis of RCTs to examine the effectiveness of MBIs in improving clinical parameters related to schizophrenia.

## 2. Method

### 2.1. Protocol and Registration

The protocol was registered in the International Prospective Register of Systematic Reviews (PROSPERO 2019: CRD42019128466) and is available at https://www.crd.york.ac.uk/prospero/display_record.php?RecordID=128466.

### 2.2. Selection Criteria

Studies were included if: (1) they were RCTs published as articles; (2) the participants were exclusively adults diagnosed with schizophrenia or related disorders (schizophrenia disorder, schizoaffective disorder, or delusional disorder) according to the two main diagnostic manuals, the Diagnostic and Statistical Manual of Mental Disorders (DSM) and the International Statistical Classification of Diseases and Related Health Problems (ICD), in their different versions; (3) the intervention group consisted of an MBI in a format similar to the MBSR or MBCT program; (4) the control group consisted of a waitlist or treatment as usual (TAU); (5) the results reported about psychological variables directly or indirectly related to schizophrenia (e.g., positive and negative symptoms or depressive symptoms).

### 2.3. Sources of Information

The bibliographic search was conducted according to the protocol guidelines of the Preferred Reporting Items for Systematic Reviews and Meta-Analyses (PRISMA) [31]. The articles retrieved included all those published between the first reference available and 25 March 2020 in the databases PsycINFO, PubMed, WOS, and Cochrane Library.

### 2.4. Search

Each search used a combination of the term “mindfulness” combined with the terms “schizophrenia”, “psychosis” or “psychotic”, in the entire text. Results from the database search were then downloaded into EndNote X9 (Clarivate Analytics, Philadelphia, PA, USA) for deduplication both electronically and manually.

### 2.5. Selection of Studies

The articles’ eligibility assessment was systematically and independently conducted by the first author (R.M.H.-C.) and the second author (C.D.-S.), and disagreements were resolved in interviews between the two reviewers. The collection of articles took place in March 2020. When articles were identified that used data from the same study, only the most recently published article was included.

### 2.6. Risk of Bias Across Studies

To assess the studies’ potential bias, we used the second version of the Cochrane risk-of-bias tool for randomized trials (RoB 2) [32]. This tool allows the assessment of risk of bias arising from five domains: bias derived from the randomization process, bias due to deviations from intended interventions, bias due to missing outcome data, bias in the measurement of the outcome, and bias in the selection of the reported result. The studies’ methodological quality was decided by consensus between the assessment ratings of the first author (R.M.H.-C.) and those of the second author (C.D.-S.).

### 2.7. Summary of Results

The estimation of treatment effects on the dependent variables, for each study, was based on the calculation of the standardized mean change using raw score standardization (SMCR; see Appendix A), taking the standard deviations of the pretest [33,34]. Bias correction was applied to the effect sizes obtained [35]. Interpretation of effect sizes follows Cohen’s recommendations [36], with values 0.20, 0.50, and 0.80 indicating small, medium, and large effect sizes, respectively. When a study yielded several measurements for the same psychological construct, only one effect size was calculated applying aggregation method [37], using the MAd package [38] for R (R Foundation for Statistical Computing, Vienna, Austria) [39].

Due to the expected heterogeneity, a random-effects model was estimated employing a restricted maximum likelihood method (REML) [40]. This procedure allows the generalization of the findings, considering the individual studies as a random sample of the population [41]. The calculation of heterogeneity was based on Cochran’s Q test, supplemented with the I2 index and was considered null with values of 0%, mild with values of 25%, moderate with values of 50%, and high with values of 75% [42]. For each variable considered, a random-effects model was implemented when three or more studies were available.

A meta-regression analysis was conducted to analyze the potential moderator variables chosen when there were five or more available studies [43]. The variables considered in the meta-regression analysis were adherence to treatment, methodological quality measured with the Revised Cochrane risk-of-bias tool for randomized trials (RoB 2), duration of treatment, type of therapy, control group, age, and gender. In order to provide more robustness to the findings obtained, a sensitivity analysis was conducted, employing three alternative models of meta-analysis chosen according to their robustness [41,44]. The publication bias was assessed when there were five or more studies, through the inspection of funnel plots, together with Egger’s test [45]. In the cases were previous appraisals had detected publication bias, it was corrected employing the trim-and-fill method (TaF) [46]. The radar charts allowed us to verify the model adjustment. Once the effect sizes were obtained, calculations for taking the omnibus test were conducted using the R package meta [47] for R [39].

## 3. Results

### 3.1. Study Selection

**Figure 1:**
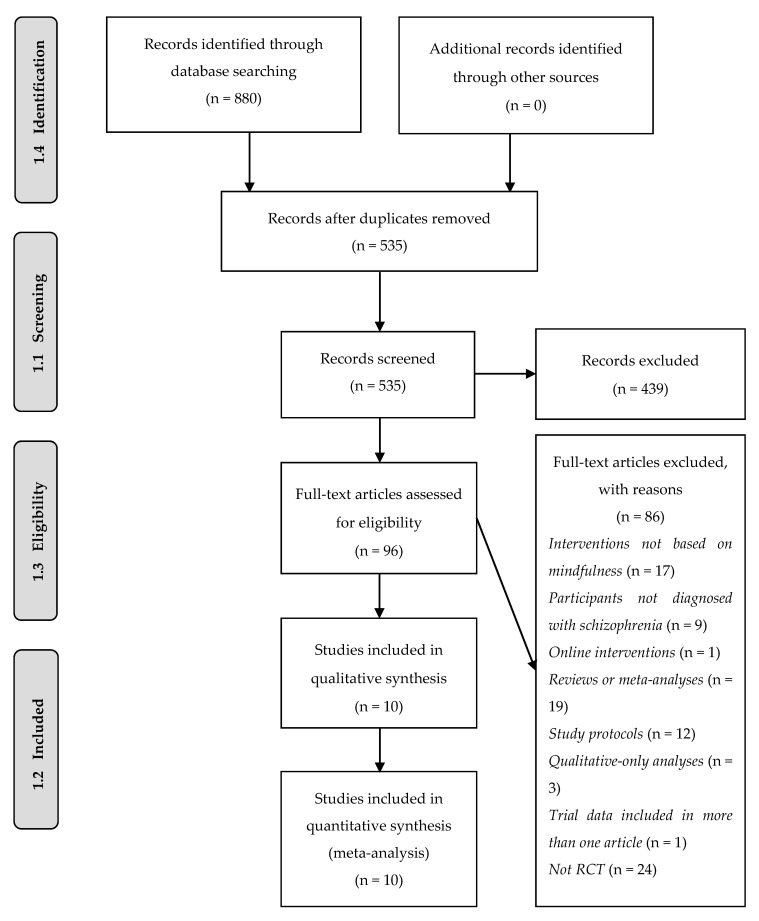
Search and selection of articles in PRISMA flow diagram.

The database search (see Figure 1) identified a total of 535 potentially relevant bibliographic records, which were then screened for possible inclusion. The studies were assessed against the mentioned inclusion and exclusion criteria, resulting in 525 discarded articles and a total of 10 articles eligible for review.

### 3.2. Study Characteristics

The general characteristics of the studies included in the review can be consulted in Table S1 of the Supplementary Materials.

### 3.3. Design of the Studies

Three studies conducted pretest and posttest measurements [48,49,50]; the rest of studies conducted pretest, posttest, and follow-up measurements. These follow-up measurements took place at different time points, which varied from 24 to 96 weeks (mean = 50 weeks). Two different types of control conditions were identified: (a) active treatments of a psychological type: psychoeducational or social support interventions (n = 272) and (b) non-psychological standard interventions (treatment as usual, TAU): medical-pharmacological treatments (n = 402).

### 3.4. Participants

The participants of the 10 studies (n = 1094) were all adults (mean age = 35.7; standard deviation = 7.9) with a diagnosis of schizophrenia, schizophreniform disorder, schizoaffective disorder, or delusional disorder according to the following diagnostic manuals: ICD-10 [51], DSM-IV [52], DSM-IV-TR [53], or DSM-5 [54]. There was a majority of male participants (60.4%). Overall, 420 participants (38.4%) were involved in some type of MBI.

### 3.5. Interventions

We found a variety of implemented MBIs in the reviewed studies (see Supplementary Materials, Table S1); however, in all of them, the core element of the intervention consisted of mindfulness training of the participants, accompanied by psychoeducation on various topics and group exercises of different nature. Additionally, in all studies, the MBI was applied alongside the participants’ TAU at the time of the study. In the studies by Chien and colleagues [55,56,57], Lee [58], Wang et al. [59], and Yilmaz and Kavak [50], MBIs were based on the MBSR program and included psychoeducational contents for people with schizophrenia. The MBIs in the studies by Chadwick and colleagues [48,60] and Langer et al. [49] were based on the MBSR program and included elements of PBCT [18]. Davis et al. [61], on the other hand, combined the MBI with a “vocational rehabilitation program”, which consisted of the inclusion of participants in paid work (20 h/week), as well as guidance and assistance provided by a vocational rehabilitation counselor on a monthly basis.

### 3.6. Risk of Bias of the Studies

Table S2 of the Supplementary Materials provides a summary of the studies’ quality. Five studies were given an assessment of low risk of bias, two an assessment of some concerns of bias, and three an assessment of high risk of bias.

### 3.7. Summary of Results

#### 3.7.1. Effects on Overall Symptomatology (Pretest–Posttest)

**Figure 2:**
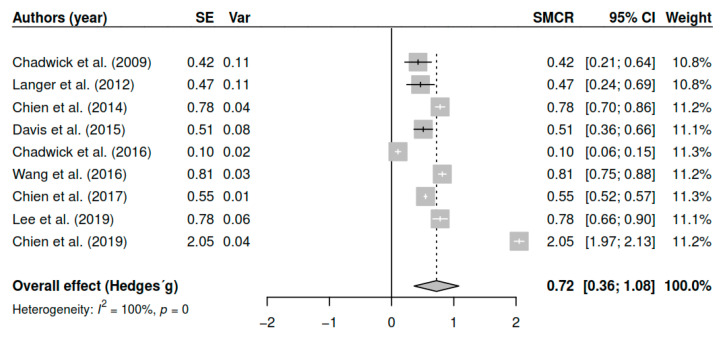
Forest plot for the model of random-effects and the values of Hedges’ g statistics in the overall symptomatology variable (pretest–posttest).

The results obtained regarding overall symptomatology (k = 9; n = 726) in the posttest measurements show statistically significant differences with moderate to large effect sizes [g = 0.72, *p* < 0.01, CI 95% (0.36, 1.08)]. Figure 2 shows the effect sizes for the results of individual studies and global effects. The heterogeneity analysis shows a high variability [I2 = 99.60, Q (8) = 1910.8, *p* < 0.01].

#### 3.7.2. Effects on Overall Symptomatology (Follow-Up)

**Figure 3:**
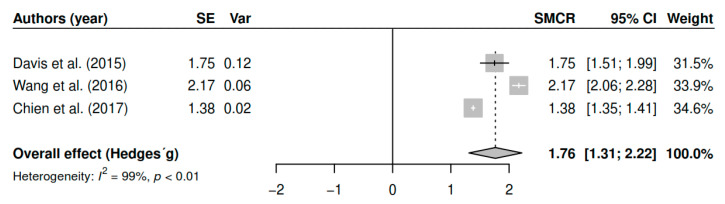
Forest plot for the model of random-effects and the values of Hedges’ g statistics in the overall symptomatology variable (follow-up).

The results obtained regarding overall symptomatology (k = 3; n = 344) in the measurements of the 6-month follow-up have shown statistically significant results with high effect sizes [g = 1.76, *p* < 0.01, CI 95% (1.31, 2.22)]. Figure 3 shows the effect sizes for the results of individual studies and global effects. The heterogeneity analysis shows a high variability [I2 = 98.90, Q (2) = 187.72, *p* < 0.01].

#### 3.7.3. Effects on Positive Symptoms (Pretest–Posttest)

**Figure 4:**
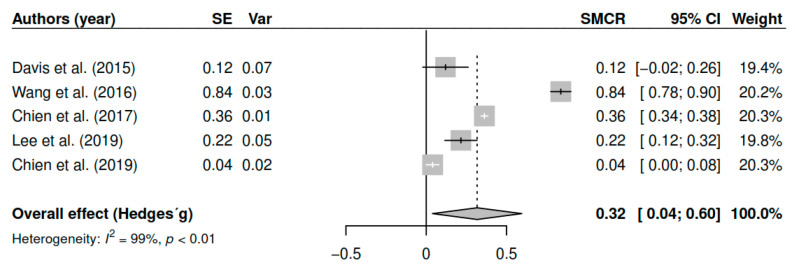
Forest plot for the model of random-effects and the values of Hedges’ g statistics in the positive symptoms variable (pretest–posttest).

The results obtained regarding positive symptoms (k = 5; n = 506) in the posttest measurements show statistically significant differences with small to moderate effect sizes [g = 0.32, *p* = 0.03, CI 95% (0.04, 0.59)]. Figure 4 shows the effect sizes for the results of individual studies and global effects. The heterogeneity analysis shows a high variability [I2 = 99.20, Q (4) = 484.10, *p* < 0.01]. 

#### 3.7.4. Negative Symptoms (Pretest–Posttest)

**Figure 5:**
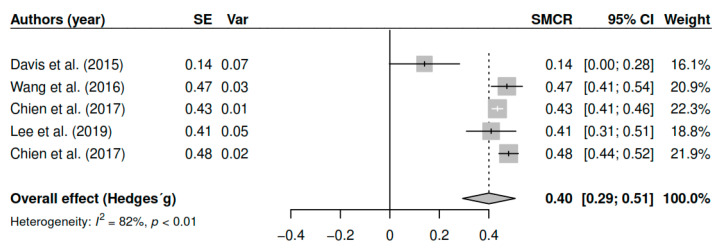
Forest plot for the model of random-effects and the values of Hedges’ g statistics in the negative symptoms variable (pretest–posttest).

The results obtained regarding the negative symptoms (k = 5; n = 506) in the posttest measurements have shown statistically significant differences with small to moderate effect sizes [g = 0.40, *p* < 0.01, CI 95% (0.29, 0.51)]. Figure 5 shows the effect sizes for the results of individual studies and global effects. The heterogeneity analysis shows a high variability [I2 = 82.50, Q (4) = 22.80, *p* < 0.01].

#### 3.7.5. Effects on Mindfulness (Pretest–Posttest)

**Figure 6:**
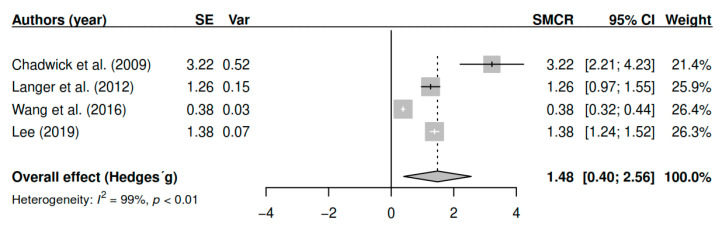
Forest plot for the model of random-effects and the values of Hedges’ g statistics in the mindfulness variable (pretest–posttest).

The results obtained regarding mindfulness abilities (k = 4; n = 178) in the posttest measurements have shown statistically significant results with large effect sizes [g = 1.48, *p* = 0.01, CI 95% (0.40, 2.56)]. Figure 6 shows the effect sizes for the results of individual studies and global effects. The heterogeneity analysis shows a high variability [I2 = 98.60, Q (3) = 220.19, *p* < 0.01].

#### 3.7.6. Effects on Functioning (Pretest–Posttest)

**Figure 7:**
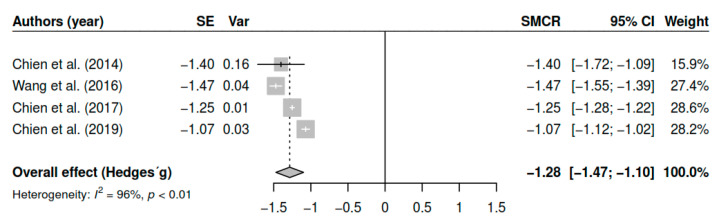
Forest plot for the model of random-effects and the values of Hedges’ g statistics in the functioning variable (pretest–posttest).

The results obtained regarding the functioning levels (k = 4; n = 493) in the posttest measurements have shown statistically significant results with large effect sizes [g = −1.28, *p* < 0.01, CI 95% (−1.47, −1.10)]. Figure 7 shows the effect sizes for the results of individual studies and global effects. The heterogeneity analysis shows a high variability [I2 = 95.90, Q (3) = 73.55, *p* < 0.01].

#### 3.7.7. Effects on Awareness of Illness (Pretest–Posttest)

**Figure 8:**
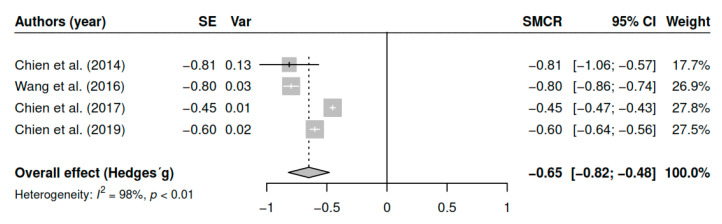
Forest plot for the model of random-effects and the values of Hedges’ g statistics in the awareness of illness variable (pretest–posttest).

The results obtained regarding awareness of illness (k = 4; n = 493) in the posttest measurements have shown statistically significant results with moderate effect sizes [g = −0.65, *p* < 0.01, CI 95% (−0.82, −0.48)]. Figure 8 shows the effect sizes for the results of individual studies and global effects. The heterogeneity analysis shows a high variability [I2 = 97.80, Q (3) = 135.18, *p* < 0.01].

### 3.8. Additional Analyses

#### 3.8.1. Results of the Meta-Regression

Table 1 shows the results of the meta-regression analysis regarding the overall symptomatology (pretest–posttest) and positive and negative symptoms with the following moderating variables: age, gender, adherence to treatment, methodological quality, specific treatment protocol, duration of the treatment, and type of control group. The results obtained show significant effects of the moderating variable duration on positive symptoms as well as of the variables age, sex, and methodological quality on negative symptoms. In this regard, we considered relevant the fact that the study by Davis et al. [61] had a small sample of participants in which there was a high percentage of men compared to women and in which the average age was high relative to the rest of the studies included in this meta-analysis. For this reason, we performed a sensitivity analysis excluding this study (see Supplementary Materials, Table S3). As can be seen in this new analysis, the moderating variables no longer have a significant effect on positive and negative symptoms.

#### 3.8.2. Sensitivity Analysis

Table 2 shows the results of fitting the random effects meta-analysis model using three methods of effect size estimation chosen for their robustness [40,44]. The results obtained with these three methods lead to similar results in all the variables analyzed.

#### 3.8.3. Publication bias and model adjustment

**Figure 9:**
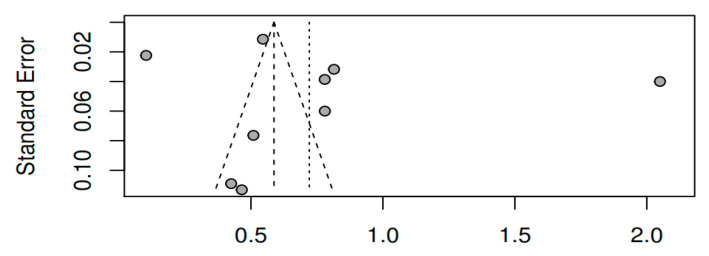
Funnel plot of the overall symptomatology variable (pretest–posttest).

**Figure 10:**
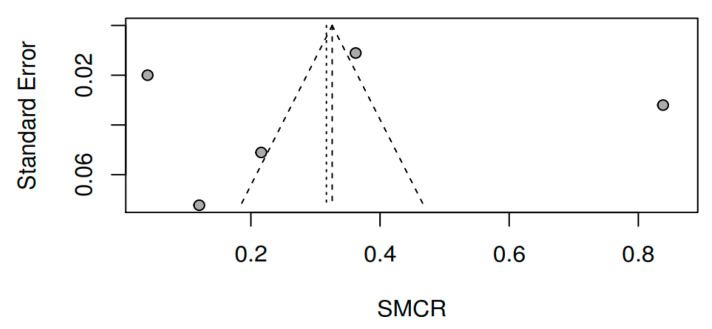
Funnel plot of the positive symptoms variable (pretest–posttest).

**Figure 11:**
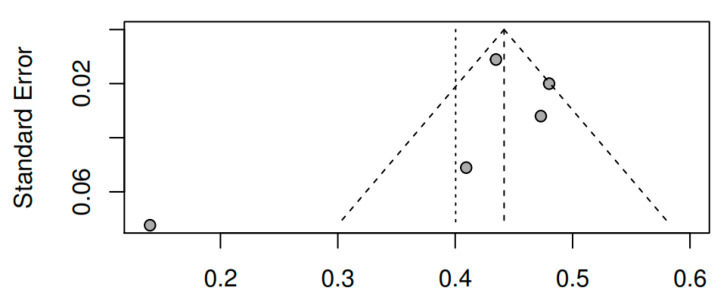
Funnel plot of the negative symptoms variable (pretest–posttest).

**Figure 12:**
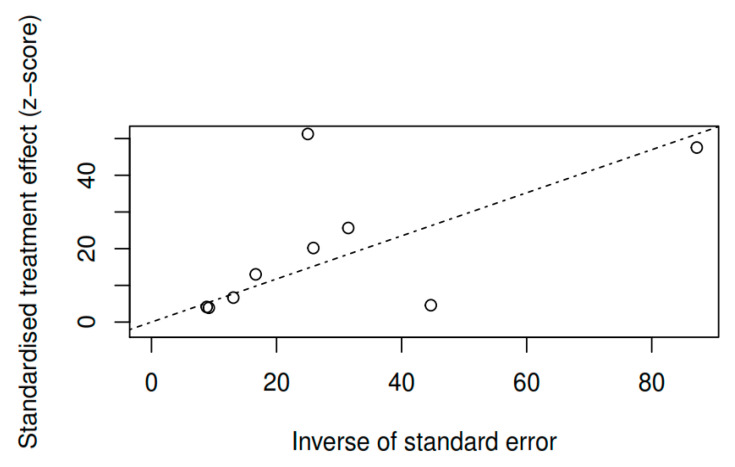
Radial adjustment chart of the random effects model for the variable overall symptomatology (pretest–posttest).

**Figure 13:**
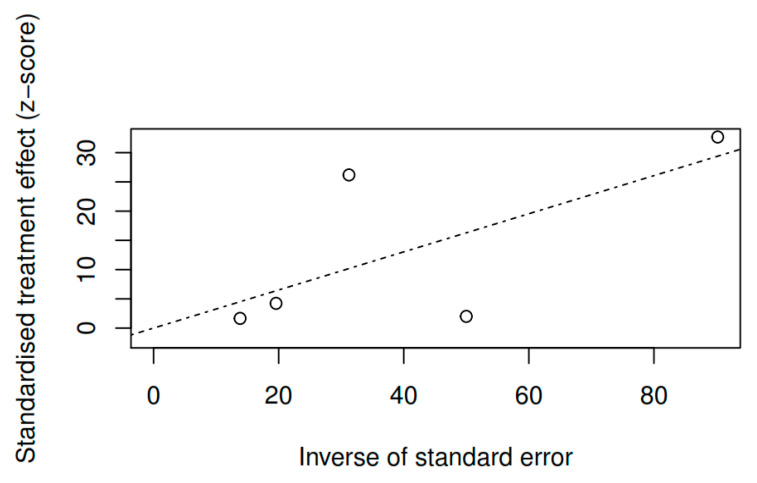
Radial adjustment chart of the random effects model for the variable positive symptoms (pretest–posttest).

**Figure 14:**
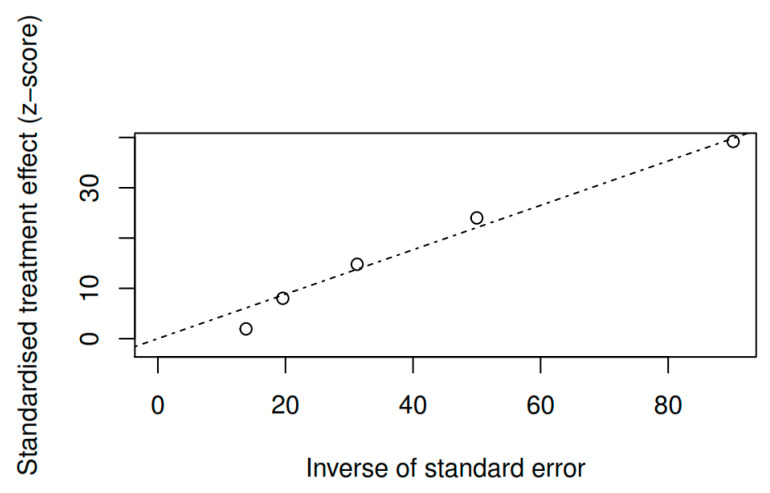
Radial adjustment chart of the random effects model for the variable negative symptoms (pretest–posttest).

When assessing publication bias by visual inspection of the funnel plots for pretest–posttest measures of the variable overall symptomatology (see Figure 9), together with Egger’s test [t (7) = 0.77, *p* = 0.46], the results obtained do not indicate that they are influenced by publication bias. For the variables positive symptoms [t (3) = −0.01, *p* = 0.99] and negative symptoms [t (3) = −0.81, *p* = 0.48], the results also do not indicate publication bias (see Figure 10 and Figure 11). As for the remaining variables, since five or more studies were not reported, the corresponding bias analysis was not performed. Concerning model fit, Figure 12, Figure 13 and Figure 14 illustrate the radial versions of the forest plot, in which certain fit problems are shown for the variables psychotic symptoms and negative symptoms. However, assuming the use of robust methods such as those contemplated in the sensitivity analysis (see Table 2), and whose results are similar to the method used here, we can assume the robustness of the REML method in the face of the violation of normality assumptions.

## 4. Discussion

The present systematic review and meta-analysis included ten studies and a total of 1094 participants. The results showed that MBIs combined with TAU are effective for the treatment of schizophrenia when compared with both TAU control groups and active treatment control groups—mostly psychoeducation groups—under the same time and frequency conditions as the MBIs. The MBIs generated moderate to large effects in reducing overall symptomatology and small to moderate effects in reducing both positive and negative symptoms in pretest–posttest comparisons. The results suggested that these effects were not mediated by the variables age, gender, adherence to treatment, methodological quality, specific treatment protocol, duration of the treatment, or type of control group.

Another relevant result is that the MBIs generated large magnitude effects on the psychosocial functioning level and moderate magnitude effects on the level of disease awareness. However, the data for these results came from four studies in which the MBIs included psychoeducational elements aimed explicitly at their improvement. Despite this, we consider that this result is noteworthy from a clinical point of view since improvements in the levels of psychosocial functioning and awareness of illness are highly relevant objectives in the treatment and recovery of people with schizophrenia [62,63].

Moreover, although only four studies analyzed changes in the mindfulness variable in the pretest–posttest comparisons, all of them reported large magnitude changes. This result is relevant since it points to a possible relationship between mindfulness and the therapeutic changes found in these trials after the application of MBIs. Future studies should explore this matter further.

Overall, the findings of this review differed from those reported by the four meta-analyses published to date on the effectiveness of interventions that include elements of mindfulness for people with psychosis [27,28,29,30]. In the reviews by Cramer et al. [28], Louise et al., [29] and Jansen et al. [30], treatments produced small to moderate effects on overall symptomatology, which is in line with our findings; Khoury et al. [27] did not analyze this outcome. Cramer et al. [28] and Jansen et al. [30], again in line with our findings, reported small to moderate effects of MBIs on positive symptoms; however, in Khoury et al. [27] and Louise et al. [29], the changes in this variable did not reach significance. Regarding negative symptoms, only Jansen et al. [30] reported—in agreement with our findings—small but significant effects. In any case, due to the methodological differences already discussed, caution must be exercised when comparing the results presented in this review with those of the mentioned meta-analyses.

An interesting finding of this review was that, although MBIs provided, on the whole, better clinical results than psychoeducation groups, this advantage was not usually reflected in the posttest measurements but rather in the follow-up measurements, six months after the end of the intervention. In the scrutiny of the data, it was be observed that this result was not so much due to MBIs’ higher retention capacity of therapeutic effects but rather to a steady increase of their effects over time. In this regard, it was observed that MBIs generated greater effects on overall symptomatology in the comparisons between the pretest and follow-up measurements (moderate–large effects) than in the comparisons between the pretest and posttest measurements (large effects). We believe this finding could provide a future line of research.

None of the studies reviewed reported any harmful effects related to the implementation of MBIs, which suggests that this type of intervention is safe for people with schizophrenia when structured protocols of intervention are followed. Thus, the present review did not find empirical evidence to support the risks suggested in previous investigations [16,64] regarding the possible exacerbation of psychotic symptoms as a result of mindfulness training. The reviewed trials shared certain elements of mindfulness practice which may have a positive impact on their therapeutic safety: (a) application within the context of a structured protocol, (b) training facilitated by instructors, (c) group practice, and (d) short-duration session practice (4 of the 9 studies specify that the duration of the practice sessions was between 10 and 30 minutes). A recent study has, in fact, found that these three factors—structured protocol, group, and short-duration practice—were associated with a lower occurrence of the unwanted side effects linked to mindfulness practice in the general population [65].

The effects obtained from MBIs in this review were comparable to those obtained in meta-analytic studies regarding the effectiveness of CBT in the reduction of positive and negative symptoms [23,66,67]. Nevertheless, the effectiveness of MBIs in the reduction of psychotic symptoms is noteworthy, given that the objective of these interventions is not to modify this type of symptoms—an aspect on which CBT does tend to focus [68,69]—but rather to manage the psychological discomfort they may cause by modifying the relationship that the patient establishes with them. On the other hand, this review found an average dropout rate in MBIs of 15.6%, similar to the dropout rate reported in efficacy trials of CBT, such as the one conducted by Burns et al. [22], where it averaged 14%. However, a recent meta-analysis on the efficacy of low-intensity CBT (<16 contact hours) reported a considerably lower dropout rate of 5.53% [23]. Now, considering these results, in which both therapeutic approaches—MBIs and CBT—are similar in terms of efficacy and safety, the choice between one or the other could be based—as suggested in a previous review [70]—on factors concerning their effectiveness in terms of cost and time. In both senses, MBIs tend to be highly efficient because they are applied to groups, unlike CBT which tends to be applied to individual patients [23] as recommended by relevant clinical practice guidelines for the psychosocial treatment of schizophrenia [71,72].

Despite the relevance of the described findings, they must be considered with caution, given the limitations of the present review. In five of the ten studies, we found certain aspects that affect their methodological quality (see Supplementary Materials, Table S2) and which, therefore, threaten the validity of their findings. Additionally, consideration should be given to the high degree of heterogeneity that we found in the analyses and which constitutes a source of bias. This heterogeneity could be related to the differences between the applied MBI protocols. Although the applied MBIs shared mindfulness practice as the core element and guiding theme of the interventions and by extension the change of relationship with psychotic symptoms, they were heterogeneous regarding the psychoeducational contents which are, in fact, active ingredients that interfere with the results’ interpretation, so that they cannot be attributed to mindfulness with absolute certainty. On the other hand, the studies were conducted in a variety of geographic locations with sample participants from different ethnic backgrounds, which is also a considerable source of heterogeneity. In this regard, four of the reviewed studies [55,56,57,59] used samples exclusively made up of recently diagnosed volunteers—in the last five years—which can affect the possibilities of generalizing the results—e.g., to include individuals who feel little motivation toward the intervention or who have suffered the illness for a longer period of time.

## 5. Conclusions

The results of this review suggest that MBIs are effective in the treatment of people with schizophrenia spectrum disorders when used as adjuvant to TAU, so that they may in the near future become one of the chosen psychosocial approaches to treat people with psychosis, together with the interventions currently recommended in clinical guides.

However, it is also clear that the empirical evidence available at present is scarce; it is, therefore, necessary to conduct further studies that fulfill methodological quality standards while analyzing sample sizes that ensure the potential validity of results in order to draw sound conclusions on the effectiveness of MBIs. The results so far are promising.

## Figures and Tables

**Table 1:** Analysis of moderating variables (meta-regression).

Variable	*β*	*z* ^1^	*se*	95% CI	*p*
Overall symptomatology					
Age	<−0.01	−0.74	0.32	[−0.02; 0.01]	0.46
Gender	<0.01	−0.17	0.01	[−0.01; 0.01]	0.86
Duration	<0.01	1.29	0.01	[−0.01; 0.03]	0.20
Treatment	0.14	1.79	0.08	[−0.01; 0.29]	0.07
Quality	0.05	0.47	0.11	[−0.16; 0.26]	0.64
Adherence	−0.01	−0.92	0.01	[−0.04; 0.02]	0.36
Control group	0.23	1.37	0.17	[−0.10; 0.55]	0.17
Positive symptoms					
Age	−0.01	−0.63	0.01	[−0.04; 0.02]	0.53
Gender	0.01	0.86	0.01	[−0.01; 0.02]	0.39
Duration	0.03	2.31	0.01	[0.01; 0.06]	0.02 *
Treatment	0.05	0.18	0.26	[−0.46; 0.55]	0.86
Quality	−0.12	−0.80	0.15	[−0.42; 0.18]	0.42
Adherence	−0.04	−1.63	0.03	[−0.09; 0.01]	0.10
Control group	0.12	0.31	0.40	[−0.67; 0.92]	0.76
Negative symptoms					
Age	−0.01	−2.01	0.01	[−0.01; <−0.01]	0.04 *
Gender	0.01	2.47	0.01	[<0.01; 0.01]	0.01 *
Duration	0.01	1.08	0.01	[−0.01; 0.02]	0.28
Treatment	0.12	1.46	0.08	[−0.04; 0.29]	0.14
Quality	−0.08	−2.05	0.04	[−0.16; −0.01]	0.04 *
Adherence	−0.01	−0.85	0.01	[−0.03; 0.01]	0.40
Control group	−0.02	−0.09	0.17	[−0.34; −0.31]	0.92

^1^ Wald’s test. * *p* < 0.05.

**Table 2:** Sensitivity analysis.

Variable	Estimator	Hedges’ *g*	95% CI	*p*
Overall symptomatology				
(Pretest–posttest)	DL	0.72	[0.38; 1.05]	<0.01 *
	ML	0.72	[0.39; 1.06]	<0.01 *
	EB	0.72	[0.36; 1.08]	<0.01 *
Overall symptomatology				
(Follow-up)	DL	1.76	[1.18; 2.35]	<0.01 *
	ML	1.76	[1.39; 2.14]	<0.01 *
	EB	1.76	[1.31; 2.21]	<0.01 *
Positive symptoms				
	DL	0.32	[0.07; 0.56]	0.01 *
	ML	0.32	[0.07; 0.57]	0.01 *
	EB	0.32	[0.04; 0.59]	0.02 *
Negative symptoms				
	DL	0.42	[0.36; 0.48]	<0.01 *
	ML	0.40	[0.36; 0.48]	<0.01 *
	EB	0.40	[0.28; 0.51]	<0.01 *
Mindfulness				
	DL	1.41	[0.66; 2.15]	<0.01 *
	ML	1.45	[0.55; 2.35]	<0.01 *
	EB	1.49	[0.36; 2.61]	0.01 *
Functioning				
	DL	−1.28	[−1.44; −1.12]	<0.01 *
	ML	−1.28	[−1.44; −1.12]	<0.01 *
	EB	−1.28	[−1.46; −1.10]	<0.01 *
Awareness of illness				
	DL	−0.65	[−0.81; −0.50]	<0.01 *
	ML	−0.65	[−0.79; −0.50]	<0.01 *
	EB	−0.65	[−0.82; −0.48]	<0.01 *

Note. DL: DerSimonian-Laird. ML: Maximum Likelihood. EB: Empirical Bayes. * *p* < 0.05.

## Supplementary Materials

The following are available online at https://www.mdpi.com/1660-4601/17/13/4690/s1. Table S1: Description of the studies included in the meta-analysis. Table S2: Summary of the quality of the reviewed studies using the Cochrane Risk of Bias Tool. Table S3: Sensitivity analysis regarding moderating variables (positive and negative symptoms).

## Appendix A

Formulas used to calculate the effect sizes in the individual studies [34,43]:

(A1) $$c[df(d_{IGPP})]=1-\frac{3}{4(n_{E}+n_{C}-2)-1}$$

**Equation (A1)**. Bias correction hedges independent groups pretest–posttest.

(A2) $$(d_{IGPP})=c[df(d_{IGPP})]\frac{\left(M_{pre,E}-M_{pos,E})-(M_{pre,C}-M_{pos,C}\right)}{\bar{S_{pre}}}$$

**Equation (A2)**. Effect size independent groups pretest–posttest.

(A3) $$\sigma^{2}(d_{IGPP})=c{\left[df(d_{IGPP})\right]}^{2}2(1-r)(\frac{n_{E}+n_{C}}{n_{E}n_{C}})(\frac{n_{E}+n_{C}-2}{n_{E}+n_{C}-4})[1+\frac{n_{E}n_{C}d_{IGPP}^{2}}{2(1-r)(n_{E}+n)}]-d_{IGPP}^{2}$$

**Equation (A3)**. Variance independent groups pretest–posttest.

(A4) $$(\bar{S_{pre}})=\sqrt{\frac{(n_{E}-1)S_{pre,E}^{2}+(n_{C}-1)S_{pre,C}^{2}}{n_{E}+n_{C}-2}}$$

**Equation (A4)**: Average standard deviations in the pre-test.
