# Supplementary material for: Mindfulness-Based Interventions for People with Schizophrenia: A Systematic Review and Meta-Analysis
Source: Int J Environ Res Public Health. 2020 Jun 30;17(13):4690. doi: 10.3390/ijerph17134690 (PMC7369977; doi:10.3390/ijerph17134690)
Supplement: Supplementary file 1 [file ijerph-17-04690-s001.pdf]

Supplementary Materials

Table S1. Description of the studies included in the meta-analysis.

Study	Country	*n* (females %)	Mean age	Experimental group			Control group	Measures	Outcome measures
				Intervention	Number of sessions	Experimenta l attrition			
Chadwick et al., 2009	England	22 (NS)	41,6	PBCT + TAU. *n*= 11.	10	18%	TAU; *n*= 11.	Pretest and posttest.	Psychological global distress (CORE). Mindfulness (SMQ and SMVQ). Psychiatric symptoms (PSYRATS). Beliefs about voices (BAVQ-r).
Chadwick et al., 2016	England	108 (50)	42	PBCT + TAU. *n*= 54.	12	15%	TAU; *n*= 54.	Pretest, Posttest and 6 months.	Psychological global distress (CORE). Psychiatric symptoms (PSYRATS and HADS). Outcome for cognitive behavior therapy for psychosis (CHOICE).
Chien & Thompson, 2014	China, Hong Kong	107 (43)	25,6	MBPP + TAU. *n*= 36.	12	11%	CG1: Psychoeducation program; *n*= 36 + TAU. CG2: TAU; *n*= 35.	Pretest, Posttest, 12 months and 24 months.	Psychiatric symptoms (BPRS). Functioning (SLOF). Social support (SSQ6). Insight (ITAQ). Number and length of stay of psychiatric readmissions.
Chien et al., 2017	China, Hong Kong, Taiwan	342 (37)	25,6	MBPP + TAU. *n*= 114.	12	16%	CG1: Psychoeducation program + TAU; *n*= 114. CG2: TAU; *n*= 114.	Pretest, posttest, 12 months and 24 months.	Psychiatric symptoms (PANSS). Functioning (SLOF). Insight (ITAQ). Number and length of stay of psychiatric readmissions.
Chien et al., 2019	China, Hong Kong, Taiwan	180 (37)	34,6	MBPP + TAU. *n*= 60.	12	8%	CG1: Psychoeducation program + TAU; *n*= 60. CG2: TAU; *n*= 60.	Pretest, posttest, 9 months and 18 months.	Psychiatric symptoms (PANSS). Functioning (SLOF). Insight (ITAQ). Mindfulness (FFMQ; measured only in the EG). Number and length of stay of psychiatric readmissions.

Davis et al., 2015	United States	34 (3)	51,7	MIRRORS + TAU + vocational rehabilitation. *n*= 18.	32	9%	Vocational rehabilitation group + TAU; *n*= 40.	Pretest, posttest and 6 months.	Psychiatric symptoms (PANSS). Work performance (WBI). Openness and involvement in the change process (CAS). Mindfulness (MFS). Intervention satisfaction (CSQ-8)
Langer et al., 2012	Spain	23 (43)	34,3	MBCT + TAU. *n*= 11.	8	18%	TAU; *n*= 12.	Pretest and posttest.	Psychiatric symptoms and clinical change (CGI-SCH). Experiential avoidance (AAQ-II). Mindfulness (SMQ).
Lee, 2019	China, Taiwan	60	52,8	MBI + TAU. *n*= 30.	8	33%	TAU; *n*= 30.	Pretest, posttest and 3 months.	Mindfulness (MAAS). Psychiatric symptoms (BDI-II, PANSS and SANS).
Wang et al., 2016	China, Hong Kong	138 (48)	24,3	MBPP + TAU. *n*= 46.	12	5%	CG1: Psychoeducation program + TAU; *n*= 46. CG2: TAU; *n*= 46.	Pretest, posttest and 6 months.	Functioning (SLOF). Psychiatric symptoms (PANSS). Recovery (QPR). Insight (ITAQ). Mindfulness (FFMQ). Number and length of stay of psychiatric readmissions.
Yılmaz & Kavak, 2018	Turkey	80 (23)	≈34	MBPP + TAU. *n*= 40.	12	15%	TAU; *n*= 40.	Pretest and posttest.	Internalized stigma (ISMI).

Note. AAQ-II: Acceptance and Action Scale; BAVQ-r: Beliefs about Voices Questionnaire revised; BDI-II: Beck Depression Inventory; BPRS: Brief Psychiatric Rating Scale; CAS: Change Assessment Scale; CG: control group; CGI-SCH: Clinical Global Impression-Schizophrenia Scale; CHOICE: Choice of outcome in cognitive behavior therapy for psychosis; CORE: Clinical Outcomes in Routine Evaluation; CSQ-8: Client Satisfaction Questionnaire; EG: experimental group; FFMQ: Five Facet Mindfulness Questionnaire; HADS: Hospital Anxiety and Depression Scale; MBI: Mindfulness-based Intervention; ISMI: Internalized Stigma of Mental Illness Scale; ITAQ: Insight and Treatment Attitudes Questionnaire; MAAS: Mindfulness Attention Awareness Scale; MBCT: Mindfulness-Based Cognitive Therapy; MBPP: Mindfulness-Based Psychoeducation Program; MFS: Mindfulness Fidelity Scale; MIRRORS: Mindfulness Intervention for Rehabilitation and Recovery in Schizophrenia; NS: not specified in the article; PANSS: Positive and Negative Syndrome Scale; PBCT: Person-Based Cognitive Therapy; PSYRATS: Psychiatric Symptom Rating Scale; QPR: Questionnaire about the Process of Recovery; SANS: Scale for Assessment of Negative Symptoms; SLOF: Specific Level of Functioning Scale; SMQ: Southampton Mindfulness Questionnaire; SMVQ: Southampton Mindfulness Voices Questionnaire; SQM: Southampton Mindfulness Questionnaire; SSQ6: Six-item Social Support Questionnaire; TAU: treatment as usual; WBI: Workshop Behavior Checklist

Table S2. Summary of the quality of the reviewed studies, using the Cochrane Risk of Bias Tool. The symbols represent low risk of bias (+), unclear risk of bias (?), or high risk of bias (-).

	Randomization process	Deviations from intended interventions	Missing outcome data	Measurement of the outcome	Selection of the reported result	Overall Bias
Chadwick et al., 2009	?	+	?	+	?	!
Chadwick et al., 2016	+	+	+	+	+	+
Chien & Thompson, 2014	+	+	+	+	+	+
Chien et al., 2017	+	+	+	+	+	+
Chien et al., 2019	+	+	+	+	+	+
Davis et al., 2015	–	+	?	+	–	–
Langer et al., 2012	?	–	–	+	?	–
Wang et al., 2016	+	+	+	+	+	+
Yılmaz & Kavak, 2018	?	+	?	+	?	!
Lee, 2019	?	–	–	?	+	–

Table S3. Sensitivity analysis regarding moderating variables (positive and negative symptoms).

Variable	*β*	*z* [1]	*se*	95% CI	*p*
Positive symptoms					
Age	-0.02	-0.05	0.31	[-0.63; 0.60]	0.95
Gender	0.01	0.53	0.03	[-0.04; 0.06]	0.59
Duration	0.04	10.85	0.02	[-0.01; 0.08]	0.06
Treatment	-0.20	-0.42	0.46	[-10.11; 0.71]	0.67
Quality	-0.10	-0.42	0.23	[-0.55; 0.36]	0.67
Adherence	-0.04	-10.21	0.03	[-0.10; 0.02]	0.22
Control group	0.20	0.42	0.46	[-0.71; 10.11]	0.67
Negative symptoms					
Age	-0.01	-0.89	0.01	[-0.01; 0.01]	0.37
Gender	-0.01	-0.37	0.01	[-0.01; 0.01]	0.70
Duration	-0.01	-0.33	0.01	[-0.01; 0.01]	0.74
Treatment	-0.04	-0.82	0.06	[-0.16; 0.07]	0.41
Quality	-0.02	-0.82	0.03	[-0.08; -0.03]	0.41
Adherence	-0.01	-0.67	0.01	[-0.01; 0.01]	0.50
Control group	0.05	0.06	-0.07	[0.16; 0.16]	0.41

[1] Wald's test. * $p < 0.05$
